# Supplementary material for: Conformational changes in the catalytic region are responsible for heat-induced activation of hyperthermophilic homoserine dehydrogenase
Source: Commun Biol. 2022 Jul 14;5:704. doi: 10.1038/s42003-022-03656-7 (PMC9283420; doi:10.1038/s42003-022-03656-7)
Supplement: Supplementary file 2 — Supplementary Information [file 42003_2022_3656_MOESM2_ESM.pdf]

Supplementary Table 1. Disordered amino acid residues.

Structural model	Disordered residues	The number of disordered residues
IM (Mol A)	None	0
IM (Mol B)	183-191, 229-231	12
IM/NADP/BU (Mol A)	228-231	4
IM/NADP/BU (Mol B)	163-170, 224-232	17
M (Mol A)	None	0
M (Mol B)	186, 229-231	4
M/NAD/Cys*	227, 254	2

*The symmetric unit contains one StHSD molecule. The disordered residues are included in the flexible part (residues 160-190) and the adjacent part (residues 224-254) in the catalytic region (residues 146-255).

Supplementary Table 2. Apparent inhibition constants for NADP+ at various conditions.

Conditions		K_i (μM)*	
pH	Temperature (K)	Immature	Mature
6.0	303	65 ± 7.0	86 ±12
7.0	303	26 ± 1.7	48 ± 1.2
8.0	303	67 ±16	99 ±17
7.0	313	24 ± 1.4	41 ± 8.7
7.0	323	13 ± 1.4	22 ± 2.4

*Apparent K_i is shown.

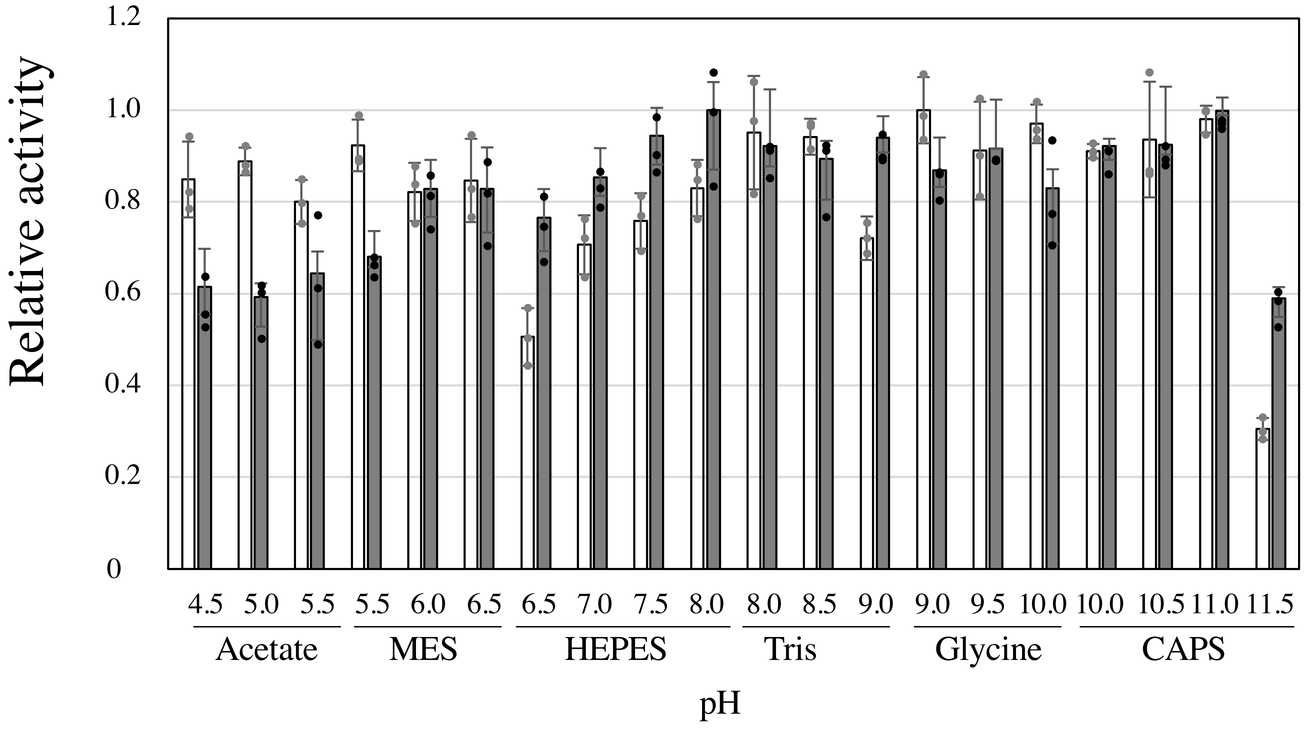

Supplementary Figure 1. pH stabilities of the enzymes.

The activity of the immature (white bar) and mature enzymes (grey bar) were incubated in 100 mM acetate (pHs 4.5, 5,0 and 5.5), MES (pHs 5.5, 6.0 and 6.5), HEPES (pHs 6.5, 7.0, 7.5 and 8.0), Tris (pHs 8.0, 8.5 and 9.0), Glycine (pHs 9.0, 9.5 and 10.0) or CAPS (pHs 10.0, 10.5, 11.0 and 11.5) at 353 K for 10 min and the residual activities were assayed in 100 mM Tris pH 8.0 at 303 K. The activity of the untreated enzyme was considered as 1.0.

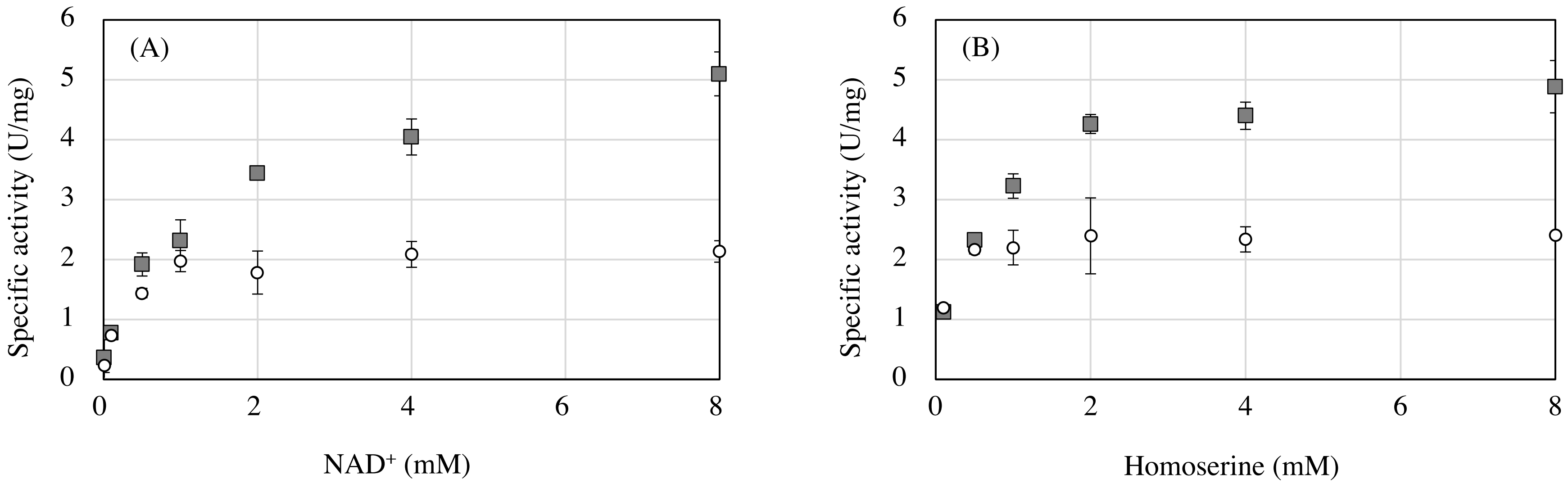

Supplementary Figure 2. Kinetics plots for V_{max} *and* K_{m} values.
The V_{max} *and* K_{m} values in Table 1 were calculated by fitting Michaelis-Menten equations. The specific activity of the immature (white) and mature enzymes (grey) were assayed in 100 mM Tris (pHs 8.0) at 303 K in the presence of 10 mM (A) homoserine or (B) NAD^+.

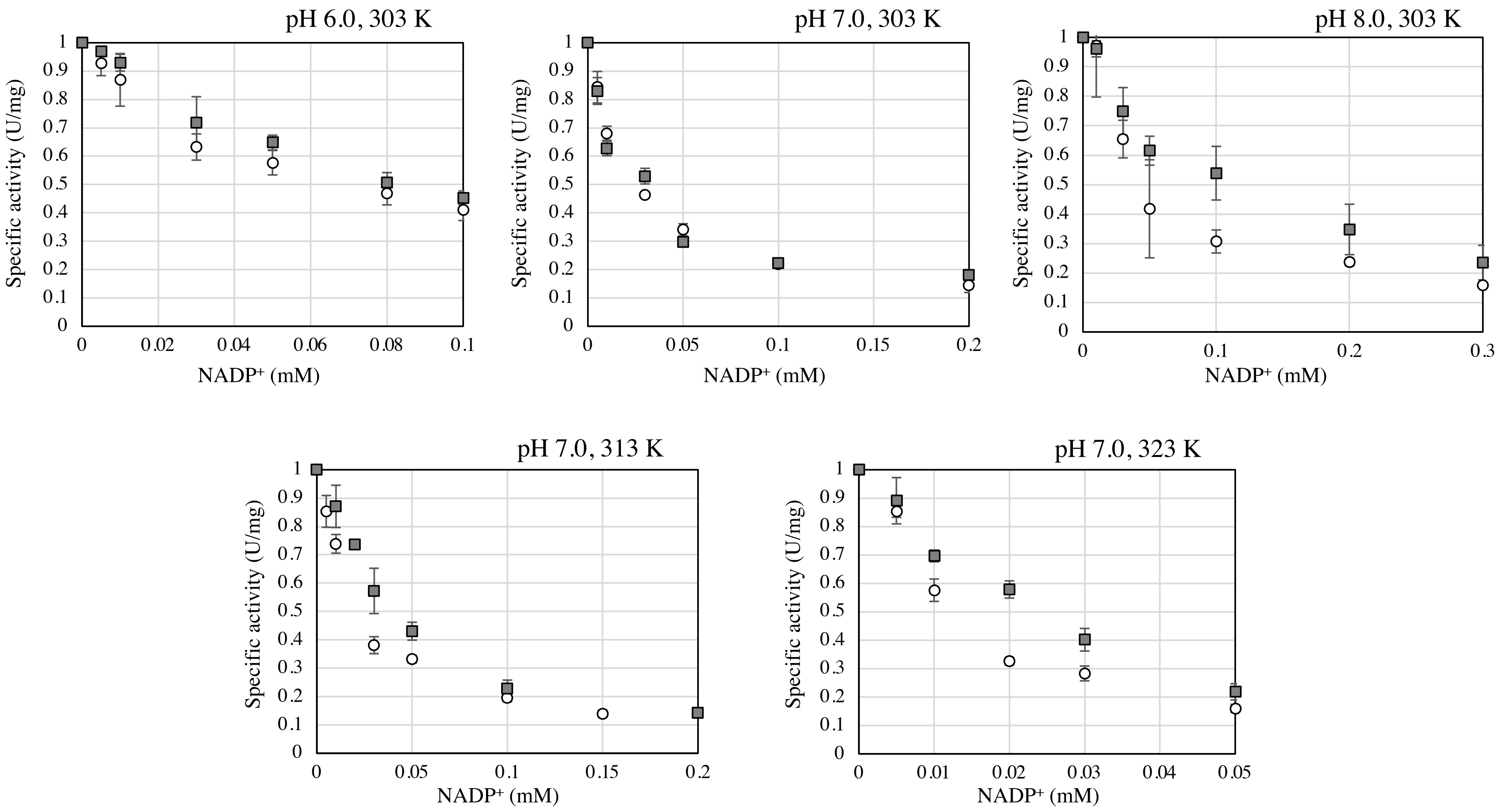

Supplementary Figure 3. Kinetics plots for calculation of apparent K_i values for $NADP^+$. The values shown in Supplementary Table 2 were calculated by fitting Morrison equations. The specific activity of the immature (white) and mature enzymes (grey) were assayed in 100 mM MES (pH 6.0), HEPES (pH 7.0) or Tris (pH 8.0) at 303 K and in 100 mM HEPES (pH 7.0) at 313 or 323 K.
